# An Enhanced Lightweight Anonymous Authentication Scheme for a Scalable Localization Roaming Service in Wireless Sensor Networks

**DOI:** 10.3390/s16101653

**Published:** 2016-10-07

**Authors:** Youngseok Chung, Seokjin Choi, Youngsook Lee, Namje Park, Dongho Won

**Affiliations:** 1 Electronics and Telecommunications Research Institute, Daejeon 34044, Korea; yschung11@nsr.re.kr (Y.C.); choisj@nsr.re.kr (S.C.); 2 Department of Computer Engineering, Sungkyunkwan University, Suwon 16419, Korea; 3 Department of Cyber Security, Howon University, Gunsan 54058, Korea; ysooklee@howon.ac.kr; 4 Department of Computer Education, Jeju National University, Jeju 63243, Korea; namjepark@jejunu.ac.kr

**Keywords:** anonymity, privacy, authentication, security, roaming service, wireless sensor network

## Abstract

More security concerns and complicated requirements arise in wireless sensor networks than in wired networks, due to the vulnerability caused by their openness. To address this vulnerability, anonymous authentication is an essential security mechanism for preserving privacy and providing security. Over recent years, various anonymous authentication schemes have been proposed. Most of them reveal both strengths and weaknesses in terms of security and efficiency. Recently, Farash et al. proposed a lightweight anonymous authentication scheme in ubiquitous networks, which remedies the security faults of previous schemes. However, their scheme still suffers from certain weaknesses. In this paper, we prove that Farash et al.’s scheme fails to provide anonymity, authentication, or password replacement. In addition, we propose an enhanced scheme that provides efficiency, as well as anonymity and security. Considering the limited capability of sensor nodes, we utilize only low-cost functions, such as one-way hash functions and bit-wise exclusive-OR operations. The security and lightness of the proposed scheme mean that it can be applied to roaming service in localized domains of wireless sensor networks, to provide anonymous authentication of sensor nodes.

## 1. Introduction

**Figure 1:**
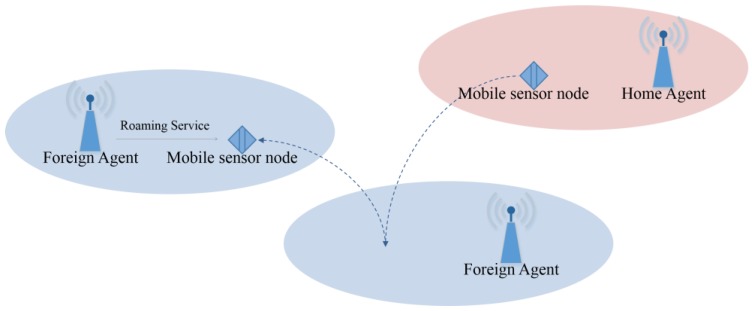
Simplified model of wireless sensor networks for roaming service.

Privacy protection and security provision have been of great concern in proportion to the number of sensor nodes in wireless sensor networks. In addition, due to the features of wireless environments, efficiency is one noticeable aspect. The characteristics of low transmission bandwidth, insufficient memory, low computing power, and battery dependency demand more lightweight and efficient security mechanisms that provide a similar level of security to wired environments. Considering a mobile sensor node that travels in various networks and wants to receive roaming service from a foreign agent, an anonymous authentication scheme is necessary to preserve the sensor node’s privacy and security. If the scheme is also lightweight, it is more suitable for wireless sensor networks. Figure 1 illustrates a simple model of wireless sensor networks for roaming service. If a mobile sensor node registered for its home agent visits a foreign network, it wants to access the foreign agent to receive roaming service. The foreign agent then needs to check the identification of the sensor node through its home agent. In this situation, a lightweight anonymous authentication scheme is necessary to guarantee secure authentication and efficient communication.

In recent years, various anonymous authentication schemes and related protocols in wireless networks have been proposed [1,2,3,4,5,6,7,8,9,10,11,12,13,14,15,16,17,18,19,20,21,22,23,24,25]. They have been followed by proofs of vulnerability of the schemes and associated improvements. Some of these schemes use high-cost functions, such as symmetric cryptographic functions, asymmetric cryptographic functions, and modular operations [1,2,3,4,5,6,7,8,9,10,11,12,13,14,15,16,17,18]. On the other hand, the others are based on low-cost functions, such as one-way hash functions and bit-wise exclusive-OR operations [19,20,21,22,23,24,25]. To analyze these schemes, we categorize them into two groups according to the computation cost: schemes based on high-cost functions and schemes based on low-cost functions. If all of them provide the same security level, schemes based on low-cost functions are more suitable for wireless sensor networks, since they consume less energy. Farash et al. [18] proposed one of the most recent anonymous authentication schemes for roaming service. They claimed that their scheme improved security and reduced computation time. However, their scheme still has security weaknesses, and does not have computational benefit.

The contributions of this paper are two points. Firstly, we point out that Farash et al.’s scheme does not provide anonymity against a legitimate but malicious adversary, foreign agent authentication, or password replacement. In addition, we present that Farash et al.’s scheme has less computational merits than our proposed scheme, even if their scheme is superior to other previous schemes in terms of the computation cost. Secondly, we propose an enhanced lightweight anonymous authentication scheme that resolves the above weaknesses. Our proposed scheme has the advantage of security and efficiency. In other words, it has enhanced security features and resistance against well-known attacks, as well as the fastest running time among other schemes. More specifically, the proposed scheme preserves weak and strong anonymity, hop-by-hop authentication, and untraceability; resistance against password guessing, impersonation, forgery, and known session key attack; and fair key agreement. There have been no recent schemes, which guarantee all the above. In addition, since the proposed scheme is based only on low-cost functions, it runs faster and more efficiently than previous schemes. Although most schemes including ours are adaptable to wireless sensor networks, our proposed scheme, due to better efficiency, has superiority over other previous schemes.

The remainder of this paper is organized as follows. Section 2 briefly describes related works, and Section 3 reviews Farash et al.’s scheme. Section 4 then presents its weaknesses. Section 5 proposes our enhanced scheme, and Section 6 presents the formal analysis of our proposed protocol. Section 7 and Section 8 then analyze the security and performance of our scheme, respectively. Finally, Section 9 concludes the paper.

## 2. Related Works

Previous schemes, which have recently been proposed, show the following research trends. Zhu and Ma [1] in 2004 proposed an anonymous authentication scheme based on high-cost functions, and Lee et al. [2] proved that it has security weaknesses. Wu et al. [3] argued that both Zhu and Ma’s and Lee et al.’s schemes fail to preserve anonymity and backward secrecy, and they presented improvements of Lee et al.’s scheme. However, Lee et al. [5] and Xu [6] showed vulnerabilities of Wu et al.’s scheme. Kun et al. [7] improved Xu and Feng’s scheme, but Tsai et al. [8] showed that Kun et al.’s scheme is also vulnerable. In addition, Mun et al. [9] showed Wu et al.’s scheme suffers from various attacks, and proposed an enhanced scheme. However, Zhao et al. [10] proved that Mun et al.’s scheme is insecure.

Independently, Chang et al. [19] in 2009 proposed an enhanced authentication scheme that uses only low-cost functions. Unfortunately, Youn et al. [20] proved that Chang et al.’s scheme is vulnerable. In addition, Zhou and Xu [13] showed that Chang et al.’s scheme has weaknesses, and they proposed an improved scheme. Lately, Gope and Hwang [24] have proved that Zhou and Xu’s scheme suffers from some security faults, such as unsuccessful key agreement and vulnerability to replay attack. They showed that a malicious adversary, by replacing transmission messages, can disturb valid communication between a normal user and a foreign agent. In addition, they proved that an attacker can successfully retransmit authentication messages that have been transmitted during a previous session of communication. At the same time, they proposed an improved scheme. Their improved scheme guarantees several security features as follows. Since all participants can normally verify parameters in each message, their scheme preserves mutual authentication. The fact that each participant makes the same contribution to the freshness of a session key provides their scheme with fair key agreement. In addition, both passive eavesdroppers and active intruders cannot identify or keep track of a normal user. Since only a legitimate user can form a valid one-time-alias using a real identity, secret value, nonce, and timestamp, no attackers can forge the alias to cheat users. In addition, it is impossible to accomplish a known session key attack because there is no significant relation among any session keys. It means that the compromised session key never helps to recover any past or future session keys. Moreover, since their scheme is based on low-cost functions, it has computational merits.

Meanwhile, He et al. [21] proved that Chang et al.’s scheme has a security fault, and that their scheme is not efficient. After that, Jiang et al. [14] showed the weaknesses of He et al.’s scheme. They proposed an enhanced protocol, but Wen et al. [15] presented its weaknesses. Subsequently, Gope and Hwang [17] showed that Wen et al.’s scheme suffers from several attacks. In Wen et al.’s scheme, an attacker, by performing an exhaustive search operation of all possible values, can obtain secret information stored in the lost or stolen smart card. After jamming all transmission messages and resetting a counter, he or she can also establish a session key between a normal user and a foreign agent. Since a session key contains only one random number generated by one side of the participants, it fails to preserve fair key agreement. In addition, Gope and Hwang proposed an enhanced scheme that preserves mutual authentication, fair key agreement, user anonymity, resistance against forgery attack, and security assurance in the case of a lost smart card. In their schemes, all participants can authenticate each other by verifying parameters. While computing a session key, each participant contributes equally, by providing independent random numbers. Since the difficulty of the quadratic residue problem makes a real identity secure, the identity cannot be revealed. No attackers can forge transmission messages because they do not have the knowledge of a secret key and a real identity. In addition, an attacker cannot use the lost or stolen smart card to perform any masquerade attacks because there is no way to obtain a secret key, an identity, and a password from the smart card.

In addition, Shin et al. [16] proved He et al.’s scheme is vulnerable, and proposed an improved scheme. Then, Farash et al. simultaneously presented the vulnerabilities of both Wen et al.’s scheme and Shin et al.’s scheme, proving that Wen et al.’s scheme suffers from session key disclosure attack and known session key attack, while Shin et al.’s scheme does not guarantee untraceability, secrecy of the sensitive parameter of home agent, secrecy against impersonation attack, or session key secrecy. Farash et al. also proposed an improved scheme that preserves security and reduces the computation time of their scheme.

## 3. Review of Farash et al.’s Scheme

In this section, we review the lightweight anonymous authentication scheme proposed by Farash et al. Their scheme consists of three phases: registration, login and authentication, and password change. Three different entities are involved in each phase. MN is a mobile node that wants to receive roaming service while visiting a foreign network. FA is the foreign agent of a foreign network, and HA is the home agent of the mobile node MN. When MN visits a foreign network, it sends a login request message to FA to be authenticated. Then, FA sends an authentication request message to HA for authentication of MN, since FA is not the home agent of MN, and it cannot directly check MN’s identity. After HA authenticates MN using the message received from FA, HA sends a response message to FA. Finally, FA sends a response message to MN and shares a common session key with MN. In this process, it is supposed that HA and FA are in a trusting relationship, and that they secretly share and store a long-term secret key. Because of this, it is possible for FA to anonymously authenticate MN through HA. Table 1 denotes the notations used in this paper.

### 3.1. Registration Phase

To register for HA, MN first selects $ID_{MN}$, $PW_{MN}$, and the random number r. Then, MN sends $ID_{MN}$ and $h(PW_{MN}||r)$ to HA in a secure manner. After receiving $ID_{MN}$ and $h(PW_{MN}||r)$, HA computes the following parameters for MN:

(1) $$A_{MN}=h\left(K_{H}\right)⊕h\left(ID_{MN}\right)$$

(2) $$B_{MN}=h(K_{H}||ID_{MN})⊕h(PW_{MN}||r)$$

Next, HA sends $A_{MN}$, $B_{MN}$, and h(.) to MN; and MN stores r, as well as $A_{MN}$, $B_{MN}$, and h(.).

### 3.2. Login and Authentication Phase

MN and FA perform the login and authentication phase to achieve the following goals with the aid of HA:

- FA anonymously authenticates MN;
- MN and FA mutually authenticate each other;
- MN and FA share a session key.

**Figure 2:**
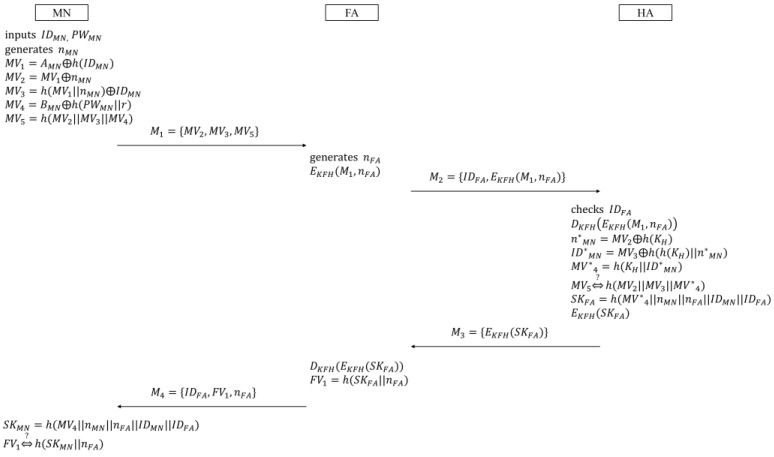
Login and authentication phase in Farash et al.’s scheme.

In this phase, it is supposed that the common secret key KFH is shared between FA and HA beforehand. Figure 2 illustrates the login and authentication phase. The procedure of this phase is as follows:

(1) MN inputs $ID_{MN}$ and $PW_{MN}$. Then MN generates the random nonce $n_{MN}$, and loads r, $A_{MN}$, $B_{MN}$, and h(.) to compute MN’s verifiers:
  (3) $$MV_{1}=A_{MN}⊕h\left(ID_{MN}\right)$$
  (4) $$MV_{2}=MV_{1}⊕n_{MN}$$
  (5) $$MV_{3}=h(MV_{1}||n_{MN})⊕ID_{MN}$$
  (6) $$MV_{4}=B_{MN}⊕h(PW_{MN}||r)$$
  (7) $$MV_{5}=h\left(MV_{2}\left|\left|MV_{3}\right|\right|MV_{4}\right)$$
  Next, MN sends the message $M_{1}=\left\{MV_{2},MV_{3},MV_{5}\right\}$ to FA.
(2) Upon receiving $M_{1}$, FA generates the random nonce $n_{FA}$, and encrypts $M_{1}$ and $n_{FA}$ using the symmetric encryption function such that $E_{KFH}\left(M_{1},n_{FA}\right)$. Then,  FA sends the message $M_{2}=\left\{ID_{FA},E_{KFH}\left(M_{1},n_{FA}\right)\right\}$ to HA.
(3) After receiving $M_{2}$, HA first checks $ID_{FA}$ to confirm that FA is a valid agent. If so, HA retrieves KFH, and makes the following computations:
  (8) $$D_{KFH}\left(E_{KFH}\left(M_{1},n_{FA}\right)\right)$$
  (9) $${n^{*}}_{MN}=MV_{2}⊕h\left(K_{H}\right)$$
  (10) $$I{D^{*}}_{MN}=MV_{3}⊕h(h\left(K_{H}\right)||{n^{*}}_{MN})$$
  (11) $$M{V^{*}}_{4}=h(K_{H}||I{D^{*}}_{MN})$$
  If HA checks the equivalence between the received $MV_{5}$ and the computed $h\left(MV_{2}\left|\left|MV_{3}\right|\right|M{V^{*}}_{4}\right)$ normally, HA computes the following session key, and encrypts it with KFH:
  (12) $$SK_{FA}=h\left(M{V^{*}}_{4}\left|\left|n_{MN}\right|\right|n_{FA}\left|\left|ID_{MN}\right|\right|ID_{FA}\right)$$
  Then, HA sends the message $M_{3}=\left\{E_{KFH}\left(SK_{FA}\right)\right\}$ to FA.
(4) After receiving $M_{3}$, FA decrypts the encrypted session key, and computes FA’s verifier:
  (13) $$FV_{1}=h(SK_{FA}||n_{FA})$$
  Then, FA sends the message $M_{4}=\left\{ID_{FA},FV_{1},n_{FA}\right\}$ to MN.
  Upon receiving $M_{4}$, MN computes the session key:
  (14) $$SK_{MN}=h\left(MV_{4}\left|\left|n_{MN}\right|\right|n_{FA}\left|\left|ID_{MN}\right|\right|ID_{FA}\right)$$
  By checking the validity of the session key after computing $h(SK_{MN}||n_{FA})$, MN confirms that FA successfully authenticates MN, and the session key is established between them at the same time.

### 3.3. Password Change Phase

**Figure 3:**
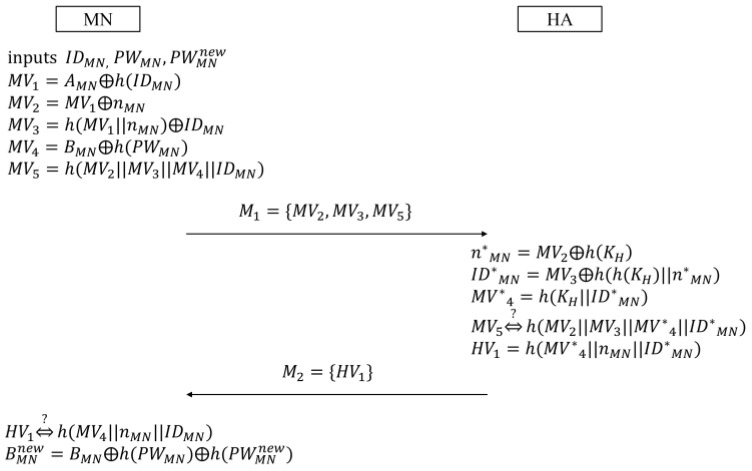
Password change phase in Farash et al.’s scheme.

MN, which wants to change its password, is supposed to perform the password change phase. In this phase, MN renews the password after acquiring the confirmation from HA. Figure 3 describes the password change phase.

(1) MN inputs the identity $ID_{MN}$, the current $PW_{MN}$, and the new password $PW_{MN}^{new}$. Then, MN generates the random nonce $n_{MN}$, and computes the following verifiers in a similar way to what it does in the login and authentication phase:
  (15) $$MV_{1}=A_{MN}⊕h\left(ID_{MN}\right)$$
  (16) $$MV_{2}=MV_{1}⊕n_{MN}$$
  (17) $$MV_{3}=h(MV_{1}||n_{MN})⊕ID_{MN}$$
  (18) $$MV_{4}=B_{MN}⊕h(PW_{MN})$$
  (19) $$MV_{5}=h(MV_{2}\left|\left|MV_{3}\right|\right|MV_{4}||ID_{MN})$$
  Next, MN sends the message $M_{1}=\left\{MV_{2},MV_{3},MV_{5}\right\}$ to HA.
(2) Upon receiving $M_{1}$, HA computes ${n^{*}}_{MN}$, $I{D^{*}}_{MN}$, and $M{V^{*}}_{4}$ as shown in Equations (20)–(22):
  (20) $${n^{*}}_{MN}=MV_{2}⊕h\left(K_{H}\right)$$
  (21) $$I{D^{*}}_{MN}=MV_{3}⊕h(h\left(K_{H}\right)||{n^{*}}_{MN})$$
  (22) $$M{V^{*}}_{4}=h(K_{H}||I{D^{*}}_{MN})$$
  After computing $h(MV_{2}\left|\left|MV_{3}\right|\right|M{V^{*}}_{4}||I{D^{*}}_{MN})$, HA checks the validity of $MV_{5}$. The successful check means HA authenticates MN normally. Then, HA computes the following verifier $HV_{1}$, and sends $M_{2}=\left\{HV_{1}\right\}$ to MN:
  (23) $$HV_{1}=h\left(M{V^{*}}_{4}\left|\left|n_{MN}\right|\right|I{D^{*}}_{MN}\right)$$
(3) After receiving $M_{2}$, MN checks the equivalence between $HV_{1}$ and $h\left(MV_{4}\left|\left|n_{MN}\right|\right|ID_{MN}\right)$ to confirm that HA has successfully authenticated MN. Finally, MN computes the following $B_{MN}^{new}$, and replaces $B_{MN}$ with $B_{MN}^{new}$:
  (24) $$B_{MN}^{new}=B_{MN}⊕h\left(PW_{MN}\right)⊕h\left(PW_{MN}^{new}\right)$$

## 4. Weaknesses of Farash et al.’s Scheme

Farash et al. proved that their scheme guarantees MN authentication, FA authentication, anonymity and untraceability, resistance against offline password guessing attack, secure key establishment, and no verification table at HA. However, there still remain several security weaknesses in their scheme. In this section, we will prove that Farash et al.’s scheme does not guarantee anonymity or FA authentication. In addition, we will show that their scheme does not achieve password replacement.

### 4.1. Anonymity

Farash et al.’s scheme guarantees anonymity against a foreign agent and a normal mobile node. However, it does not preserve anonymity against a malicious mobile node. Suppose that there is a malicious mobile node $MN^{'}$ normally registered for HA, as in Farash et al.’s attack scenario. Then, $MN^{'}$ can get $ID_{MN}$ by accomplishing the following procedures:

(1) $MN^{'}$ inputs $ID_{MN^{'}}$ and $PW_{MN^{'}}$. Then, $MN^{'}$ can get $h\left(K_{H}\right)$.
(2) To get $n_{MN}$, $MN^{'}$ eavesdrops $M_{1}=\left\{MV_{2},MV_{3},MV_{5}\right\}$, and computes $MV_{2}⊕h\left(K_{H}\right)$. Since the equation described below holds, $MN^{'}$ can successfully get $n_{MN}$:
  $$MV_{2}⊕h\left(K_{H}\right)=MV_{1}⊕n_{MN}⊕h\left(K_{H}\right)=A_{MN}⊕h\left(ID_{MN}\right)⊕n_{MN}⊕h\left(K_{H}\right)\;=h\left(K_{H}\right)⊕h\left(ID_{MN}\right)⊕h\left(ID_{MN}\right)⊕n_{MN}⊕h\left(K_{H}\right)=n_{MN}$$
(3) Next, by computing as follows, $MN^{'}$ gets $ID_{MN}$:
  $$MV_{3}⊕h(h\left(K_{H}\right)||n_{MN})=h(MV_{1}||n_{MN})⊕ID_{MN}⊕h(h\left(K_{H}\right)||n_{MN})\;=h(A_{MN}⊕h\left(ID_{MN}\right)||n_{MN})⊕ID_{MN}⊕h(h\left(K_{H}\right)||n_{MN})\;=h(h\left(K_{H}\right)⊕h\left(ID_{MN}\right)⊕h\left(ID_{MN}\right)||n_{MN})⊕ID_{MN}⊕h(h\left(K_{H}\right)||n_{MN})=ID_{MN}$$

As a result, a malicious mobile node that eavesdrops the message can easily know the other’s identity, so anonymity is not guaranteed.

### 4.2. Authentication

In Farash et al.’s scheme, HA can authenticate both MN and FA. In MN’s case, after computing $n_{MN}$ from $MV_{2}$ and $ID_{MN}$ from $MV_{3}$, HA can authenticate MN by checking the equivalence between $MV_{5}$ and $h\left(MV_{2}\left|\left|MV_{3}\right|\right|MV_{4}\right)$. In addition, HA can authenticate FA by a successful decryption using the pre-shared secret key KFH corresponding to FA’s identity. Meanwhile, FA is able to anonymously authenticate MN with the aid of HA. In addition, a successful decryption using KFH makes FA check HA’s identity. This is the same as what HA does. However, while it is possible to authenticate HA, MN cannot authenticate FA. There is no obvious way for MN to confirm the received message that is made with the aid of HA. The reason is as follows. After receiving $M_{4}=\left\{ID_{FA},FV_{1},n_{FA}\right\}$ from FA, MN just computes the following session key $SK_{MN}$, without checking any verifiers computed by HA:

$$SK_{MN}=h\left(MV_{4}\left|\left|n_{MN}\right|\right|n_{FA}\left|\left|ID_{MN}\right|\right|ID_{FA}\right)$$

Clearly, $SK_{MN}$ contains $MV_{4}$ which is computed as follows:

$$MV_{4}=B_{MN}⊕h(PW_{MN}||r)=h(K_{H}||ID_{MN})$$

Since HA is the only entity that can compute $h(K_{H}||ID_{MN})$, MN can only authenticate HA through a successful checking of $SK_{MN}$, namely $FV_{1}=h(SK_{FA}||n_{FA})$. This implies that there is no way for MN to authenticate FA. In addition, if failure while checking $FV_{1}$ occurs, MN cannot confirm whether HA or FA is illegal. For these reasons, authenticating the foreign agent is impossible.

### 4.3. Password Replacement

In the password change phase, MN computes the following $MV_{4}$, while HA computes $M{V^{*}}_{4}$:

$$MV_{4}=B_{MN}⊕h\left(PW_{MN}\right)=h(K_{H}||ID_{MN})⊕h(PW_{MN}||r)⊕h\left(PW_{MN}\right)$$

$$M{V^{*}}_{4}=h(K_{H}||I{D^{*}}_{MN})$$

Since $MV_{4}$ is not equal to $M{V^{*}}_{4}$, there is no equivalence between $MV_{5}$ and $h(MV_{2}\left|\left|MV_{3}\right|\right|M{V^{*}}_{4}||I{D^{*}}_{MN})$, as shown in:

$$MV_{5}=h(MV_{2}\left|\left|MV_{3}\right|\right|MV_{4}||ID_{MN})=h(MV_{2}\left|\left|MV_{3}\right|\right|B_{MN}⊕h\left(PW_{MN}\right)||ID_{MN})\;=\;h(MV_{2}\left|\left|MV_{3}\right|\right|h(K_{H}||ID_{MN})⊕h(PW_{MN}||r)⊕h\left(PW_{MN}\right)||ID_{MN})\;\neq h(MV_{2}\left|\left|MV_{3}\right|\right|M{V^{*}}_{4}||I{D^{*}}_{MN})=h(MV_{2}\left|\left|MV_{3}\right|\right|h(K_{H}||I{D^{*}}_{MN})||I{D^{*}}_{MN})$$

To originally authenticate MN, HA needs to confirm the validity of $MV_{5}$; but it is impossible to check this. As a result, the home agent cannot authenticate a mobile node in the password change phase, and the password replacement cannot be accomplished. Moreover, there is no HA contribution to change the password. This means that by computing $B_{MN}^{new}=B_{MN}⊕h\left(PW_{MN}\right)⊕h\left(PW_{MN}^{new}\right)$, MN can change the password as it wants, without accomplishing any other steps.

## 5. The Proposed Scheme

In this section, we propose an enhanced scheme to remedy the faults of Farash et al.’s scheme. Our scheme also consists of three phases. In each phase, MN, FA, and HA are involved, and use a timestamp as a nonce. After receiving a message, they first validate a timestamp to ensure that old messages cannot be used in replay attacks. We use the same terms as Farash et al.’s scheme does. However, to apply our scheme to wireless sensor networks, we regard MN as a mobile sensor node. Clearly, FA and HA are server systems, which have a powerful computing capability. On the other hand, MN is a battery-powered sensor node, which has less computing capability. In the registration phase, MN registers for HA, and HA gives MN secret parameters in a secure manner. MN and HA establish a trusting relationship through this phase. Then, MN roams in a foreign network, and tries to receive roaming service from FA. Since MN is not a mobile sensor node of FA, FA wants to authenticate MN through HA. For this, the login and authentication phase is necessary. It is assumed that FA and HA share a long-term secret key KFH beforehand, the same as in Farash et al.’s scheme. FA and HA are supposed to use KFH when they try to authenticate each other. Meanwhile, in the password change phase, MN, with the aid of HA, securely changes the secret key, as well as the password. Each phase is described in detail as follows.

### 5.1. Registration Phase

**Figure 4:**
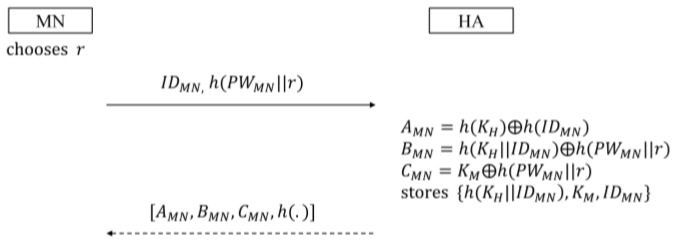
Registration phase in the proposed scheme.

The first thing MN accomplishes is to register for HA in the registration phase. Figure 4 shows this phase:

MN selects its identity $ID_{MN}$ and the password $PW_{MN}$. In addition, MN chooses the random number r as a salt of a one-way hash function. MN then submits $ID_{MN}$ and $h(PW_{MN}||r)$ to HA through a secure channel. Upon receiving the registration request message, HA, using its secret key $K_{H}$, computes three secret parameters for MN as follows:

(25) $$A_{MN}=h\left(K_{H}\right)⊕h\left(ID_{MN}\right)$$

(26) $$B_{MN}=h(K_{H}||ID_{MN})⊕h(PW_{MN}||r)$$

(27) $$C_{MN}=K_{M}⊕h(PW_{MN}||r)$$

where $K_{M}$ is the secret key allocated only to MN. Then, HA secretly stores $\left\{h(K_{H}||ID_{MN}),K_{M},ID_{MN}\right\}$ in its database, and sends $A_{MN}$, $B_{MN}$, $C_{MN}$, and h(.) to MN in a secure way. Finally, MN stores  r, $A_{MN}$, $B_{MN}$, $C_{MN}$, and h(.).

### 5.2. Login and Authentication Phase

**Figure 5:**
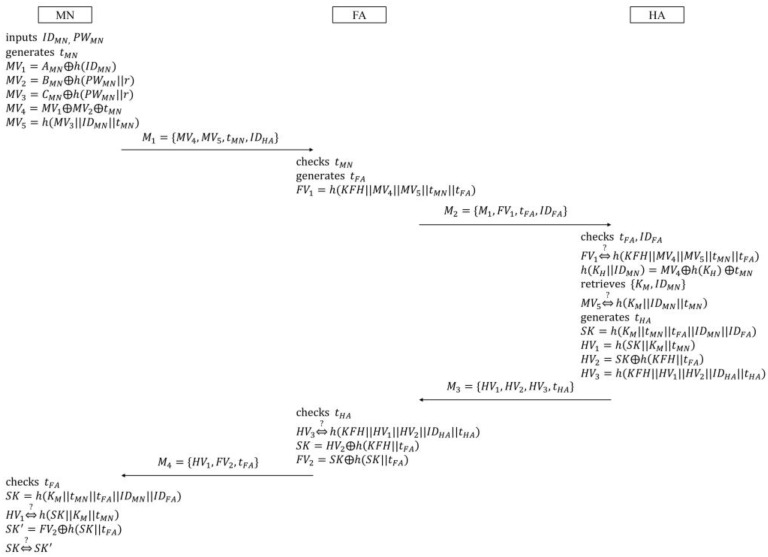
Login and authentication phase in the proposed scheme.

When MN visits a foreign network and logins to FA, FA anonymously authenticates MN through HA. MN and FA then share a session key for secure communication. Figure 5 illustrates the login and authentication phase:

(1) MN inputs $ID_{MN}$ and $PW_{MN}$ to make the login request message. Then, MN generates the timestamp $t_{MN}$, and loads r, $A_{MN}$, $B_{MN}$, $C_{MN}$, and h(.) to compute MN’s verifiers:
  (28) $$MV_{1}=A_{MN}⊕h\left(ID_{MN}\right)$$
  (29) $$MV_{2}=B_{MN}⊕(PW_{MN}||r)$$
  (30) $$MV_{3}=C_{MN}⊕(PW_{MN}||r)$$
  (31) $$MV_{4}=MV_{1}⊕MV_{2}⊕t_{MN}$$
  (32) $$MV_{5}=h\left(MV_{3}\left|\left|ID_{MN}\right|\right|t_{MN}\right)$$
  Next, MN sends the login request message $M_{1}=\left\{MV_{4},MV_{5},t_{MN},ID_{HA}\right\}$ to FA.
(2) FA, which receives $M_{1}$ from MN, first checks $t_{MN}$ to confirm whether it is valid or not. If FA confirms the validity of $t_{MN}$, FA also generates the timestamp $t_{FA}$, and computes FA’s verifier as follows:
  (33) $$FV_{1}=h\left(KFH\left|\left|MV_{4}\right|\right|MV_{5}\left|\left|t_{MN}\right|\right|t_{FA}\right)$$
  Then, FA sends the authentication request message $M_{2}=\left\{M_{1},FV_{1},t_{FA},ID_{FA}\right\}$ to HA.
(3) After receiving $M_{2}$, HA first checks $t_{MN}$ and $ID_{FA}$ to confirm whether $t_{FA}$ is valid or not, as well as whether FA is an ally or not. If HA confirms the validities of both $t_{FA}$ and $ID_{FA}$, HA fetches the secret key KFH corresponding to $ID_{FA}$, and checks the equivalence between $FV_{1}$ and $h\left(KFH\left|\left|MV_{4}\right|\right|MV_{5}\left|\left|t_{MN}\right|\right|t_{FA}\right)$. If they are equal, HA computes:
  (34) $$h(K_{H}||ID_{MN})=MV_{4}⊕h\left(K_{H}\right)⊕t_{MN}$$
  Then, HA searches $\left\{K_{MN},ID_{MN}\right\}$ from its database, using $h(K_{H}||ID_{MN})$ as a keyword. If there are no value matches with $h(K_{H}||ID_{MN})$ in the database, HA regards $M_{2}$ as a forged message. In this case, HA does not move on to the next step, and informs FA of this. Otherwise, HA checks the equivalence between $MV_{5}$ and $h\left(K_{M}\left|\left|ID_{MN}\right|\right|t_{MN}\right)$. If this is successfully verified, HA generates the timestamp $t_{HA}$, and computes the session key and HA’s verifiers:
  (35) $$SK=h\left(K_{M}\left|\left|t_{MN}\right|\right|t_{FA}\left|\left|ID_{MN}\right|\right|ID_{FA}\right)$$
  (36) $$HV_{1}=h\left(SK\left|\left|K_{M}\right|\right|t_{MN}\right)$$
  (37) $$HV_{2}=SK⊕h\left(KFH||t_{FA}\right)$$
  (38) $$HV_{3}=h(KFH\left|\left|HV_{1}\right|\right|HV_{2}\left|\left|ID_{HA}\right|\right|t_{HA})$$
  Then, HA sends the authentication response message $M_{3}=\left\{HV_{1},HV_{2},HV_{3},t_{HA}\right\}$ to FA.
(4) Upon receiving $M_{3}$, FA computes $h(KFH\left|\left|HV_{1}\right|\right|HV_{2}\left|\left|ID_{HA}\right|\right|t_{HA}$) after checking $t_{HA}$, and checks it equals $HV_{3}$. If the equality holds, FA computes the following session key and FA’s verifier, to send the login response message $M_{4}=\left\{HV_{1},FV_{2},t_{FA}\right\}$ to MN:
  (39) $$SK=HV_{2}⊕h\left(KFH||t_{FA}\right)$$
  (40) $$FV_{2}=SK⊕h(SK||t_{FA})$$
(5) After receiving $M_{4}$, MN first checks $t_{FA}$, and computes the session key:
  (41) $$SK=h\left(K_{M}\left|\left|t_{MN}\right|\right|t_{FA}\left|\left|ID_{MN}\right|\right|ID_{FA}\right)$$
  To authenticate HA, MN checks the equivalence between $HV_{1}$ and $h\left(SK\left|\left|K_{M}\right|\right|t_{MN}\right)$. If this is confirmed normally, MN obtains $SK^{'}$ by computing $FV_{2}⊕h(SK||t_{FA})$. Then, MN checks SK equals $SK^{'}$ to authenticate FA. Finally, MN and FA complete mutual authentication of each other, and share the session key between them.

### 5.3. Password Change Phase

**Figure 6:**
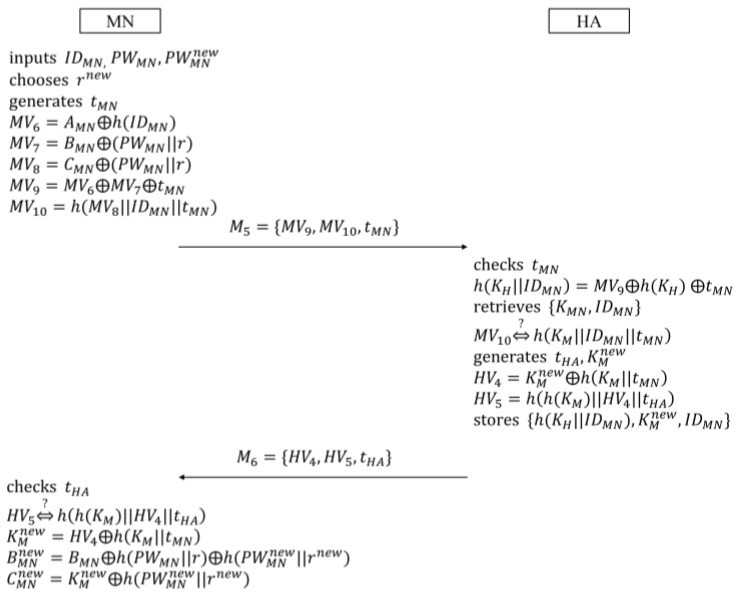
Password change phase in the proposed scheme.

In this phase, MN not only renews its password, but also the secret key. MN can change the password for itself, without being authenticated by HA. However, in order to change the secret key, it is necessary for MN to accomplish the password change procedure with HA. Figure 6 shows this phase:

(1) MN inputs its identity $ID_{MN}$, the current $PW_{MN}$, and the new password $PW_{MN}^{new}$. In addition, MN chooses the new random number $r^{new}$ as a new salt of a one-way hash function, and generates the timestamp $t_{MN}$. Then, MN computes the following verifiers in the same form as they are in the login and authentication phase:
  (42) $$MV_{6}=A_{MN}⊕h\left(ID_{MN}\right)$$
  (43) $$MV_{7}=B_{MN}⊕(PW_{MN}||r)$$
  (44) $$MV_{8}=C_{MN}⊕(PW_{MN}||r)$$
  (45) $$MV_{9}=MV_{6}⊕MV_{7}⊕t_{MN}$$
  (46) $$MV_{10}=h\left(MV_{8}\left|\left|ID_{MN}\right|\right|t_{MN}\right)$$
  Next, MN sends the message $M_{5}=\left\{MV_{9},MV_{10},t_{MN}\right\}$ to HA.
(2) HA, after receiving $M_{5}$, checks $t_{MN}$ to confirm whether it is valid or not. Then, HA computes $h(K_{H}||ID_{MN})=MV_{9}⊕h\left(K_{H}\right)\;⊕t_{MN}$. In addition, HA, using $h(K_{H}||ID_{MN})$ as a keyword, searches for $\left\{K_{MN},ID_{MN}\right\}$ from its database. If it is impossible to search $\left\{K_{MN},ID_{MN}\right\}$ due to no matching value, HA immediately stops continuing, and informs MN of this. If not, HA computes $h\left(K_{M}\left|\left|ID_{MN}\right|\right|t_{MN}\right)$, and checks that it equals $MV_{10}$. To renew the secret key $K_{M}$, HA generates the timestamp $t_{HA}$ and the new secret key $K_{M}^{new}$, and computes the following verifiers:
  (47) $$HV_{4}=K_{M}^{new}⊕h\left(K_{M}||t_{MN}\right)$$
  (48) $$HV_{5}=h\left(h(K_{M})\left|\left|HV_{4}\right|\right|t_{HA}\right)$$
  Then, after replacing $\left\{h(K_{H}||ID_{MN}),K_{M},ID_{MN}\right\}$ with $\left\{h(K_{H}||ID_{MN}),K_{M}^{new},ID_{MN}\right\}$, HA sends the message $M_{6}=\left\{HV_{4},HV_{5},t_{HA}\right\}$ to MN.
(3) Upon receiving $M_{6}$, MN validates $t_{HA}$, and then checks the equivalence between $HV_{5}$ and $h\left(h(K_{M})\left|\left|HV_{4}\right|\right|t_{HA}\right)$. If both $t_{HA}$ and $HV_{5}$ are successfully verified, MN computes the new secret key $K_{M}^{new}$ after checking :
  (49) $$K_{M}^{new}=HV_{4}⊕h\left(K_{M}||t_{MN}\right)$$
  Finally, MN computes the following secret parameters, and replaces $\left\{r,\;A_{MN},B_{MN},C_{MN},h\left(.\right)\right\}$ with $\left\{r^{new},A_{MN},B_{MN}^{new},C_{MN}^{new},h\left(.\right)\right\}$:
  (50) $$B_{MN}^{new}=B_{MN}⊕h(PW_{MN}||r)⊕h(PW_{MN}^{new}||r^{new})$$
  (51) $$C_{MN}^{new}=K_{M}^{new}⊕h(PW_{MN}^{new}||r^{new})$$

## 6. Protocol Analysis

In this section, we present the formal analysis of our proposed scheme usingBurrows–Abadi–Needham logic [26] (also known as the BAN logic), which is a useful model to prove the validity of authentication and key agreement protocol. The main goal of the login and authentication phase in our scheme is that MN and FA authenticate each other, and share a session key. Since both MN and FA participate and equally contribute while establishing a session key, it can be regarded as a two-way key agreement. In addition, in the password change phase, it is the main goal that MN and HA authenticate each other, and renew MN’s secret key. Only HA contributes while generating MN’s secret key. Therefore, renewing MN’s secret key can be regarded as a one-way key agreement.

To prove that our proposed scheme meets these goals, we need to transform the scheme into the idealized form by the analytic procedures of BAN logic. We first define the constructs and some rules of BAN logic as follows:

[Constructs]

- P|≡X: P believes X.
- P⊲X: P sees X.
- P|~X: P said X.
- P⇒X: P has jurisdiction over X.
- #(X): Formula X is fresh.
- $P\overset{K}{↔}Q$: P and Q may use the shared key K to communicate.

[Rules]

- $R_{1}$, Message-meaning rule:
  $$\frac{P|\equiv P\overset{K}{↔}Q,\;P⊲{\left\{X\right\}}_{K}}{P\left|\equiv Q\right|\sim X}$$
- $R_{2}$, Nonce-verification rule:
  $$\frac{P\left|\equiv \#\left(X\right),\;P\right|\equiv Q|\sim X}{P\left|\equiv Q\right|\equiv X}$$
- $R_{3}$, Jurisdiction rule:
  $$\frac{P\left|\equiv Q\Rightarrow X,\;P\right|\equiv Q|\equiv X}{P|\equiv X}$$
- $R_{4}$, Fresh rule:
  $$\frac{P|\equiv \#\left(X\right)}{P|\equiv \#\left(X,Y\right)}$$

Then, using BAN logic rules, we transform our goals into the following forms. The login and authentication phase needs mutual authentication and a two-way key agreement. In addition, the password change phase needs mutual authentication and a one-way key agreement.

[Transformation of the goals of the login and authentication phase]

- $G_{1}:\;MN\left|\equiv FA\right|\equiv MN\overset{SK}{↔}FA$,
- $G_{2}:FA\left|\equiv MN\right|\equiv MN\overset{SK}{↔}FA$,
- $G_{3}:MN|\equiv MN\overset{SK}{↔}FA,$
- $G_{4}:FA|\equiv MN\overset{SK}{↔}FA.$

[Transformation of the goals of the password change phase]

- $G_{5}:MN\left|\equiv HA\right|\equiv MN\overset{K_{M}^{new}}{↔}HA,$
- $G_{6}:HA\left|\equiv MN\right|\equiv MN\overset{K_{M}^{new}}{↔}HA,$
- $G_{7}:MN|\equiv MN\overset{K_{M}^{new}}{↔}HA.$

Next, the messages $M_{1}$, $M_{2}$, $M_{3}$, and $M_{4}$ in Figure 5, and $M_{5}$ and $M_{6}$ in Figure 6 are transformed into the idealized messages as follows:

[Idealized messages]

- $M_{1}:\;\left(<h(K_{H}||ID_{MN}){>}_{h\left(K_{H}\right)},{<ID_{MN}>}_{K_{M}},t_{MN}\right),$
- $M_{2}:{<<h(K_{H}||ID_{MN}){>}_{h\left(K_{H}\right)},{<ID_{MN}>}_{K_{M}},t_{FA}>}_{KFH},$
- $M_{3}:{<{<MN\overset{SK}{↔}FA>}_{\left(K_{M},t_{MN}\right)},{<MN\overset{SK}{↔}FA>}_{\left(KFH,t_{FA}\right)},t_{HA}>}_{KFH},$
- $M_{4}:\;\left({<MN\overset{SK}{↔}FA>}_{\left(K_{M},t_{MN}\right)},{<MN\overset{SK}{↔}FA>}_{\left(SK,t_{FA}\right)},t_{FA}\right),$
- $M_{5}:\;\left(<h(K_{H}||ID_{MN}){>}_{h\left(K_{H}\right)},{<ID_{MN}>}_{K_{M}},t_{MN}\right),$
- $M_{6}:{<{<MN\overset{K_{M}^{new}}{↔}HA>}_{\left(K_{M},t_{MN}\right)},t_{HA}>}_{K_{M}}$.

In addition, we make the following assumptions to analyze our proposed scheme.

[Assumptions]

- $A_{1}:\;MN|\equiv \#\left(t_{X}\right)$, where X is FA or HA,
- $A_{2}:\;FA|\equiv \#\left(t_{X}\right)$, where X is MN or HA,
- $A_{3}:\;HA|\equiv \#\left(t_{X}\right)$ , where X is MN or FA,
- $A_{4}:\;MN|\equiv MN\overset{h\left(K_{H}\right)}{\Leftrightarrow }HA,$
- $A_{5}:\;HA|\equiv MN\overset{h\left(K_{H}\right)}{\Leftrightarrow }HA,$
- $A_{6}:\;MN|\equiv MN\overset{K_{M}}{\Leftrightarrow }HA,$
- $A_{7}:\;HA|\equiv MN\overset{K_{M}}{\Leftrightarrow }HA,$
- $A_{8}:\;FA|\equiv FA\overset{KFH}{\Leftrightarrow }HA,$
- $A_{9}:\;HA|\equiv FA\overset{KFH}{\Leftrightarrow }HA$,
- $A_{10}:\;MN|\equiv HA\Rightarrow MN\overset{SK}{↔}FA,$
- $A_{11}:\;FA|\equiv HA\Rightarrow MN\overset{SK}{↔}FA,$
- $A_{12}:\;MN|\equiv MN\overset{SK}{↔}FA,$
- $A_{13}:\;HA|\equiv MN\Rightarrow ID_{MN},$
- $A_{14}:\;MN|\equiv HA\Rightarrow MN\overset{K_{M}^{new}}{↔}HA,$
- $A_{15}:\;ID_{MN}$ is unknown for anyone except MN.

Using the above rules and assumptions, we analyze the idealized form of our proposed scheme. The following procedure shows how the proposed scheme meets the goals described above:

(1) We apply $R_{4}$ and $A_{2}$ to $M_{1}$ to derive the following statement:
  $\left(S_{1}\right)$ $$FA|\equiv \#\left(<h(K_{H}||ID_{MN}){>}_{h\left(K_{H}\right)},{<ID_{MN}>}_{K_{M}}\right)$$
(2) We apply $R_{1}$ and $A_{9}$ to $M_{2}$ to derive
  $\left(S_{2}\right)$ $$HA\left|\equiv FA\right|\sim \left(<h(K_{H}||ID_{MN}){>}_{h\left(K_{H}\right)},{<ID_{MN}>}_{K_{M}},t_{FA}\right)$$
(3) We apply $R_{2}$ and $A_{3}$ to $S_{2}$ to derive
  $\left(S_{3}\right)$ $$HA\left|\equiv FA\right|\equiv \left(<h(K_{H}||ID_{MN}){>}_{h\left(K_{H}\right)},{<ID_{MN}>}_{K_{M}}\right)$$
(4) To break conjunctions, we apply the rule of BAN logic to $S_{3}$, then get
  $\left(S_{4}\right)$ $$HA\left|\equiv FA\right|\equiv <h(K_{H}||ID_{MN}){>}_{h\left(K_{H}\right)}$$
  $\left(S_{5}\right)$ $$HA\left|\equiv FA\right|\equiv {<ID_{MN}>}_{K_{M}}$$
(5) We apply $R_{1}$ and $A_{5}$ to $S_{4}$ to derive
  $\left(S_{6}\right)$ $$HA\left|\equiv MN\right|\equiv h(K_{H}||ID_{MN})$$
(6) We apply $R_{1}$ and $A_{7}$ to $S_{5}$ to derive
  $\left(S_{7}\right)$ $$HA\left|\equiv MN\right|\equiv ID_{MN}$$
(7) We apply $R_{3}$ and $A_{13}$ to $S_{7}$ to derive
  $\left(S_{8}\right)$ $$HA|\equiv ID_{MN}$$
(8) From $A_{15}$ and $S_{8}$, we can deduct the following rule:
  $\left(S_{9}\right)$ $$HA|\equiv MN\overset{ID_{MN}}{\Leftrightarrow }HA$$
(9) From $S_{6}$ and $S_{9}$, we can also deduct the following rule:
  $\left(S_{10}\right)$ $$HA\left|\equiv MN\right|\equiv MN\overset{SK}{↔}FA$$
(10) We apply $R_{1}$ and $A_{8}$ to $M_{3}$ to derive
  $\left(S_{11}\right)$ $$FA\left|\equiv HA\right|\sim \left({<MN\overset{SK}{↔}FA>}_{\left(K_{M},t_{MN}\right)},{<MN\overset{SK}{↔}FA>}_{\left(KFH,t_{FA}\right)},t_{HA}\right)$$
(11) We apply $R_{2}$ and $A_{2}$ to $S_{11}$ to derive
  $\left(S_{12}\right)$ $$FA\left|\equiv HA\right|\equiv \left({<MN\overset{SK}{↔}FA>}_{\left(K_{M},t_{MN}\right)},{<MN\overset{SK}{↔}FA>}_{\left(KFH,t_{FA}\right)}\right)$$
(12) To break conjunctions, we apply the rule of BAN logic to $S_{12}$, then get
  $\left(S_{13}\right)$ $$FA\left|\equiv HA\right|\equiv {<MN\overset{SK}{↔}FA>}_{\left(K_{M},t_{MN}\right)}$$
  $\left(S_{14}\right)$ $$FA\left|\equiv HA\right|\equiv {<MN\overset{SK}{↔}FA>}_{\left(KFH,t_{FA}\right)}$$
(13) We apply $R_{1}$, $R_{2}$, and $A_{8}$ to $S_{14}$ to derive
  $\left(S_{15}\right)$ $$FA\left|\equiv HA\right|\equiv MN\overset{SK}{↔}FA$$
(14) From $S_{10}$ and $S_{15}$, we can imply the following statement:
  $\left(S_{16}\right)$ $$FA\left|\equiv MN\right|\equiv MN\overset{SK}{↔}FA$$

In this step, we achieve $G_{2}$.

(15) We apply $R_{3}$ and $A_{11}$ to $S_{15}$ to derive
  $\left(S_{17}\right)$ $$FA|\equiv MN\overset{SK}{↔}FA$$

In this step, we achieve $G_{4}$.

(16) We apply $R_{2}$, $A_{1}$, and $A_{10}$ to $M_{4}$ to derive
  $\left(S_{18}\right)$ $$MN\left|\equiv HA\right|\equiv \left({<MN\overset{SK}{↔}FA>}_{\left(K_{M},t_{MN}\right)},{<MN\overset{SK}{↔}FA>}_{\left(SK,t_{FA}\right)}\right)$$
(17) To break conjunctions, we apply the rule of BAN logic to $S_{18}$, then get
  $\left(S_{19}\right)$ $$MN\left|\equiv HA\right|\equiv {<MN\overset{SK}{↔}FA>}_{\left(K_{M},t_{MN}\right)}$$
  $\left(S_{20}\right)$ $$MN\left|\equiv HA\right|\equiv {<MN\overset{SK}{↔}FA>}_{\left(SK,t_{FA}\right)}$$
(18) We apply $R_{1}$, $R_{2}$, $A_{1}$, and $A_{6}$ to $S_{19}$ to derive
  $\left(S_{21}\right)$ $$MN\left|\equiv HA\right|\equiv MN\overset{SK}{↔}FA$$
(19) We apply $R_{1}$, $R_{2}$, $A_{1}$, and $A_{12}$ to $S_{20}$ to derive
  $\left(S_{22}\right)$ $$MN\left|\equiv FA\right|\equiv MN\overset{SK}{↔}FA$$

In this step, we achieve $G_{1}$.

(20) We apply $R_{3}$ and $A_{10}$ to $S_{21}$ to derive
  $\left(S_{23}\right)$ $$MN|\equiv MN\overset{SK}{↔}FA$$

In this step, we achieve $G_{3}$.

(21) We apply $R_{2}$, $A_{3}$, and $A_{14}$ to $M_{5}$ to derive
  $\left(S_{24}\right)$ $$HA\left|\equiv MN\right|\equiv \left(<h(K_{H}||ID_{MN}){>}_{h\left(K_{H}\right)},{<ID_{MN}>}_{K_{M}}\right)$$
(22) To break conjunctions, we apply the rule of BAN logic to $S_{24}$, then get
  $\left(S_{25}\right)$ $$HA\left|\equiv MN\right|\equiv <h(K_{H}||ID_{MN}){>}_{h\left(K_{H}\right)}$$
  $\left(S_{26}\right)$ $$HA\left|\equiv MN\right|\equiv {<ID_{MN}>}_{K_{M}}$$
(23) We apply $R_{1}$ and $A_{5}$ to $S_{25}$ to derive
  $\left(S_{27}\right)$ $$HA\left|\equiv MN\right|\equiv h(K_{H}||ID_{MN})$$
(24) We apply $R_{1}$ and $A_{7}$ to $S_{26}$ to derive
  $\left(S_{28}\right)$ $$HA\left|\equiv MN\right|\equiv ID_{MN}$$
(25) We apply $R_{3}$ and $A_{13}$ to $S_{28}$ to derive
  $\left(S_{29}\right)$ $$HA|\equiv ID_{MN}$$
(26) From $A_{15}$ and $S_{29}$, we can deduct the following rule:
  $\left(S_{30}\right)$ $$HA|\equiv MN\overset{ID_{MN}}{\Leftrightarrow }HA$$
(27) From $S_{27}$ and $S_{30}$, we can also deduct the following rule:
  $\left(S_{31}\right)$ $$HA\left|\equiv MN\right|\equiv MN\overset{K_{M}^{new}}{↔}HA$$

In this step, we achieve $G_{6}$.

(28) We apply $R_{1}$ and $A_{6}$ to $M_{6}$ to derive
  $\left(S_{32}\right)$ $$MN\left|\equiv HA\right|\sim \left({<MN\overset{K_{M}^{new}}{↔}HA>}_{\left(K_{M},t_{MN}\right)},t_{HA}\right)$$
(29) We apply $R_{2}$ and $A_{1}$ to $S_{32}$ to derive
  $\left(S_{33}\right)$ $$MN\left|\equiv HA\right|\equiv {<MN\overset{K_{M}^{new}}{↔}HA>}_{\left(K_{M},t_{MN}\right)}$$
(30) We apply $R_{1}$, $R_{2}$, and $A_{6}$ to $S_{33}$ to derive
  $\left(S_{34}\right)$ $$MN\left|\equiv HA\right|\equiv MN\overset{K_{M}^{new}}{↔}HA$$

In this step, we achieve $G_{5}$.

(31) We apply $R_{3}$ and $A_{14}$ to $S_{34}$ to derive
  $\left(S_{35}\right)$ $$MN|\equiv MN\overset{K_{M}^{new}}{↔}HA$$

In this step, we achieve $G_{7}$.

As a result, $S_{16}$, $S_{17}$, $S_{22}$, and $S_{23}$ accomplish the goals of the login and authentication phase, and $S_{31}$, $S_{34}$, and $S_{35}$ accomplish the goals of the password change phase. By this fact, our proposed scheme preserves mutual authentication and a session key establishment between MN and FA, and mutual authentication and a secret key renewal between MN and HA.

## 7. Security Analysis

The proposed scheme guarantees anonymity, hop-by-hop authentication, untraceability, resistance against password guessing attack, resistances against impersonation and forgery attacks, resistance against known session key attack, and fair key agreement. We define two different anonymity preservations in this paper. One is weak anonymity preservation against a passive adversary who accomplishes a passive attack, like eavesdropping. The other is strong anonymity preservation against a valid but malicious node. Clearly, a malicious node is more powerful than a passive adversary because it possesses a valid sensor. If a scheme guarantees strong anonymity, it also absolutely preserves weak anonymity. The detail analysis of our scheme is described below.

### 7.1. Strong Anonymity

Among the transmission messages, only $MV_{4}$ and $HV_{5}$ contain MN’s identity $ID_{MN}$, which is formed as:

$$MV_{4}=MV_{1}⊕MV_{2}⊕t_{MN}=A_{MN}⊕h\left(ID_{MN}\right)⊕B_{MN}⊕h(PW_{MN}||r)⊕t_{MN}\;=h\left(K_{H}\right)⊕h(K_{H}||ID_{MN})⊕t_{MN}\;HV_{5}=h\left(K_{M}\left|\left|ID_{MN}\right|\right|t_{MN}\right)$$

An adversary who refers to a malicious sensor node can know $h\left(K_{H}\right)$ and $t_{MN}$. Clearly, a valid sensor node can provide $h\left(K_{H}\right)$, while $M_{1}$ can reveal $t_{MN}$. Thus, it is easy for the adversary to know $h\left(K_{H}\right)$ and $t_{MN}$. However, although knowing $h\left(K_{H}\right)$ and $t_{MN}$, there is no way to get $ID_{MN}$ from $MV_{4}$ and $HV_{5}$. This is because $ID_{MN}$ is one of the input parameters of a one-way hash function, and it is always used with the secret key $K_{H}$ or $K_{M}$. Namely, only the entity who knows $K_{H}$ or $K_{M}$ can obtain $ID_{MN}$. As a result, the proposed scheme guarantees strong anonymity against a malicious sensor node.

### 7.2. Hop-by-Hop Authentication

In the login and authentication phase, each entity, MN, FA, and HA, needs to authenticate the others. Trusting relationships between MN and HA, and FA and HA make it possible for them to check each other’s identities. First, after computing $SK=h\left(K_{M}\left|\left|t_{MN}\right|\right|t_{FA}\left|\left|ID_{MN}\right|\right|ID_{FA}\right)$, MN can authenticate HA, by checking $HV_{1}=h\left(SK\left|\left|K_{M}\right|\right|t_{MN}\right)$. Since $K_{M}$ is only known to MN and HA, a successful verification of $HV_{1}$ implies HA normally computes SK and $HV_{1}$. MN can also authenticate FA, by checking $FV_{2}=SK⊕h(SK||t_{FA})$ with the verified SK. HA is another entity other than FA that can compute $FV_{2}$, but there is no reason for HA to compute $FV_{2}$ instead of FA. This means that only FA can compute a valid $FV_{2}$. Second, by verifying $HV_{3}=h\left(KFH\left|\left|HV_{1}\right|\right|HV_{2}\left|\left|ID_{HA}\right|\right|t_{HA}\right)$, FA can authenticate HA, since KFH is a securely pre-shared secret key between FA and HA. In addition, FA can anonymously authenticate MN through HA. Although FA has no information related to MN, FA can authenticate MN, by confirming that HA ensures the identification of MN. Lastly, HA can authenticate FA, through verifying $FV_{1}=h\left(KFH\left|\left|MV_{4}\right|\right|MV_{5}\left|\left|t_{MN}\right|\right|t_{FA}\right)$. For the same reason as FA, HA can identify FA, due to KFH. Since MN is the only entity to compute $MV_{5}$ using $K_{M}$ and $ID_{MN}$, checking $MV_{5}=h\left(K_{M}\left|\left|ID_{MN}\right|\right|t_{MN}\right)$ makes HA authenticate MN. As a result, the proposed scheme provides hop-by-hop authentication among MN, FA, and HA, while they accomplish the login and authentication phase.

### 7.3. Untraceability

If there are transmission messages that have the same value throughout several sessions, an adversary can trace those messages, and know all the messages that originate from one sensor node. However, since they always contain different timestamps, every transmission message in the proposed scheme is unique in each session. In addition, an adversary cannot link two or more different sessions of the same sensor node. Therefore, the proposed scheme preserves the freshness and untraceability of every message in every session.

### 7.4. Resistance Against Password Guessing Attack

An adversary can eavesdrop any transmission messages, but there is no way to get the sensor node’s password from those messages. The reason is that no transmission messages contain the password itself, or even related information. Even if the secret parameters and a salt stored in the sensor node are revealed, it is still impossible to obtain the password. An adversary can generate a lookup table to make pre-computed hash values with candidate passwords and salt. However, changing salts in the password change phase makes it impossible to generate pre-computed hash values. Therefore, in the proposed scheme, the possibility to verify the correctness of a guessed password does not exist, and a password guessing attack is impossible.

### 7.5. Resistance Against Impersonation and Forgery Attacks

If an adversary can compute $MV_{5}$ formed as $h\left(K_{M}\left|\left|ID_{MN}\right|\right|t_{MN}\right)$, he or she is able to impersonate MN, by sending a valid login and authentication request message. However, this is absolutely impossible, since the adversary cannot know $K_{M}$ and $ID_{M}$. Meanwhile, suppose that the adversary makes the following forged message $M_{1}=\left\{M{V_{4}}^{'},M{V_{5}}^{'},{t_{MN}}^{'},ID_{HA}\right\}$, and sends it to HA via FA:

$$M{V_{4}}^{'}=h\left({K_{H}}^{'}\right)⊕h({K_{H}}^{'}||ID_{adv})⊕t_{adv}\;M{V_{5}}^{'}=h\left(K_{adv}\left|\left|ID_{adv}\right|\right|t_{adv}\right)$$

where $ID_{adv}$ is the identity of the adversary, $t_{adv}$ is the timestamp generated by the adversary, $K_{adv}$ is the secret key of the adversary, and ${K_{H}}^{'}$ is the fake secret key of HA. Then, after computing $M{V_{4}}^{'}⊕h\left({K_{H}}^{'}\right)⊕t_{adv}$, HA tries to search $h({K_{H}}^{'}||ID_{adv})$ in its database. Unfortunately, HA finds no matching value, and then it recognizes the fact that the adversary sent $M{V_{4}}^{'}$.

### 7.6. Resistance Against Known Session Key Attack

Even if the session key established between MN and FA is revealed, there is no way to compute the next session key, using the exchanged messages, $HV_{1}$ and $HV_{2}$. To compute the session key, it is necessary to know the sensor node’s secret key $K_{M}$ or the long-term secret key KFH, which is shared between FA and HA. Clearly, an adversary cannot compute the session key, since he or she does not know $K_{M}$ or KFH. Moreover, since every session key contains unique timestamps, they have no relation with each other. For this reason, the proposed scheme is resistant against known session key attack.

### 7.7. Fair Key Agreement

When MN, FA, and HA perform the login and authentication phase, the session key contains two timestamps generated by MN and FA, respectively. This implies that MN and FA make the same contribution to the freshness and randomness of the session key. In other words, both MN and FA contribute equally during the establishment of the session key. As a result, the proposed scheme achieves fair key agreement.

## 8. Security and Performance Comparisons

In this section, we compare security and performance of our scheme with the previous schemes of Jiang et al., Wen et al., Shin et al., Gope and Hwang, and Farash et al. To analyze security of each scheme, we apply the following security features to them:

- SF1: Weak anonymity,
- SF2: Strong anonymity,
- SF3: Hop-by-hop authentication,
- SF4: Untraceability,
- SF5: Resistance against password guessing attack,
- SF6: Resistance against impersonation and forgery attack,
- SF7: Resistance against known session key attack,
- SF8: Fair key agreement,
- SF9: No verification table.

In addition, we apply the experiment result of Li et al. [4] to analyze performance. The following notations show the execution times of each operation:

- H: Execution time of a one-way hash function (1H ≈ 0.0005 s),
- S: Execution time of a symmetric operation (1S ≈ 0.0087 s),
- E: Execution time of a modular exponential operation (1E ≈ 0.522 s),

To describe concisely, we also use the following terms:

- P1: Phase of login and authentication,
- P2: Phase of password change.

Table 2 denotes the security comparison of each scheme. Table 2 shows that our proposed scheme provides more enhanced security than previous schemes do. However, the schemes of Wen et al. and Shin et al. and our scheme need to maintain a verification table. The verification table stored in HA contains information for user authentication. Namely, it contains identity/counter pairs in Wen et al.’s scheme, identity/password pairs in Shin et al.’s scheme, and identity/secret key pairs in our scheme. Looking up this information takes time. However, considering HA’s strong computational power, it is negligible.

Meanwhile, Table 3 compares the computation cost in the login and authentication phase. Our proposed scheme, which is based only on low-cost functions, needs the lowest computation cost among all schemes. Table 4 shows the performance comparison in the password change phase. In the schemes of Jiang et al., Wen et al., and Gope and Hwang, MN changes his or her password without any help of HA. Whereas, the schemes of Shin et al., Farash et al., and our scheme require that both MN and HA participate while updating MN’s password. Clearly, the schemes that MN performs the password change phase alone have a little bit better efficiency. However, the computation cost of the password change phase in each scheme is slightly different, and thus it does not affect the performance of all over the scheme. Table 5 shows the total computation cost of each scheme. Consequently, as shown in Table 5, our proposed scheme runs the fastest and has the highest efficiency.

## 9. Conclusions

In this paper, we first prove that Farash et al.’s scheme fails to guarantee strong anonymity, foreign agent authentication, or password replacement. To remedy these weaknesses, we propose an enhanced security authentication scheme. The secret key for each sensor node and the password by hashing with a different salt enhance the security of our scheme. By comparing our scheme with other recent schemes, we show that it is more secure from various aspects. In addition, to reduce the computation time, our scheme only uses low-cost functions. Performance comparison shows that, as compared with the previous ones, our proposed scheme provides better lightness. This means that it provides better efficiency. Consequently, the proposed scheme is more suitable for battery-powered sensors and wireless sensor networks.

## Figures and Tables

**Table 1:** Notations.

Notation	Description
HA	Home agent
FA	Foreign agent
MN	Mobile node
$ID_{X}$	Identity of an entity X
$PW_{MN}$	Password of MN
KFH	Pre-shared secret key between HA and FA
$K_{X}$	Secret key of an entity X
$n_{X}$	Random nonce generated by an entity X
$t_{X}$	Timestamp generated by an entity X
$E_{K}\left(.\right)/D_{K}\left(.\right)$	Symmetric encryption and decryption using a secret key K
h(.)	Collision free one-way hash function
||	Concatenation
⊕	Bit-wise exclusive-OR operation

**Table 2:** Security comparison.

Scheme	SF1	SF2	SF3	SF4	SF5	SF6	SF7	SF8	SF9
Jiang et al.	Yes	Yes	No	Yes	No	Yes	Yes	Yes	Yes
Wen et al.	Yes	Yes	No	Yes	No	Yes	No	No	No
Shin et al.	Yes	Yes	No	Yes	No	Yes	Yes	Yes	No
Gope and Hwang	Yes	Yes	Yes	Yes	No	Yes	Yes	Yes	Yes
Farash et al.	Yes	No	No	Yes	No	Yes	Yes	Yes	Yes
Ours	Yes	Yes	Yes	Yes	Yes	Yes	Yes	Yes	No

**Table 3:** Performance comparison in login and authentication phase.

Scheme	MN	FA	HA	Total	
Jiang et al.	4H + 1E	4H	5H + 1E	13H + 2E	≈ 1.0505 s
Wen et al.	4H + 1E	4H + 1E	5H + 2E	13H + 4E	≈ 2.0945 s
Shin et al.	5H	1H + 2S	3H + 2S + 1E	9H + 4S + 1E	≈ 0.5613 s
Gope and Hwang	6H + 1E	3H + 1S	7H + 1S + 1E	16H + 2S + 2E	≈ 1.0694 s
Farash et al.	6H	1H + 2S	5H + 2S	12H + 4S	≈ 0.0408 s
Ours	6H	4H	7H	17H	≈ 0.0085 s

**Table 4:** Performance comparison in password change phase.

Scheme	MN	FA	HA	Total	
Jiang et al.	2H	N/A	N/A	2H	≈ 0.0010 s
Wen et al.	2H	N/A	N/A	2H	≈ 0.0010 s
Shin et al.	4H	N/A	1H + 1E	5H + 1E	≈ 0.5245 s
Gope and Hwang	2H	N/A	N/A	2H	≈ 0.0010 s
Farash et al.	6H5	N/A	5H	11H	≈ 0.0055 s
Ours	6H	N/A	5H	11H	≈ 0.0055 s

**Table 5:** Total computation cost comparison.

Scheme	P1	P2	Total	
Jiang et al.	13H + 2E	2H	15H + 2E	≈ 1.0515 s
Wen et al.	13H + 4E	2H	15H + 4E	≈ 2.0955 s
Shin et al.	9H + 4S + 1E	5H + 1E	14H + 4S + 2E	≈ 1.0858 s
Gope and Hwang	16H + 2S + 2E	2H	18H + 2S + 2E	≈ 1.0704 s
Farash et al.	12H + 4S	11H	23H + 4S	≈ 0.0463 s
Ours	17H	11H	28H	≈ 0.0140 s
